# A Facile Approach for the Ligand Free Synthesis of Biocompatible Upconversion Nanophosphors

**DOI:** 10.3389/fchem.2022.904676

**Published:** 2022-05-31

**Authors:** Elizabeth Shiby, Kumbam Lingeshwar Reddy, Jatish Kumar

**Affiliations:** Department of Chemistry, Indian Institute of Science Education and Research (IISER) Tirupati, Tirupati, India

**Keywords:** upconversion nanophosphors, ligand free synthesis, two/multi-photon absorption, biocompatible nanoparticles, anti-Stokes shift

## Abstract

Upconversion nanophosphors, particles that can absorb low energy radiation and emit high energy light through multi-photon absorption processes, have gained augmented attention in recent years. Due to their admirable optical and chemical properties, these nanoparticles are finding wide range of applications in the field of bioimaging, light emitting devices and security printing. However, for any practical application, it is extremely important that a facile synthetic route is developed that can lead to the generation of nanophosphors exhibiting efficient upconversion luminescence under diverse experimental conditions. Herein, we report a new ligand-free approach for the synthesis of lanthanide-based upconversion nanoparticles by adopting a simple solid-state synthetic route. The reaction conditions such as temperature and time were optimized to obtain nanophosphors exhibiting enhanced upconversion luminescence. The synthesized nanoparticles, due to its ligand-free nature, could be well dispersed in both aqueous and organic media. The nanophosphors retained the upconversion luminescence under varying time, pH and temperature, indicating that the absence of ligand had least effect on their stability. The nanophosphors were found to exhibit good cell viability even under high concentrations, unveiling their potential as bioimaging agents in the biomedical research.

## Introduction

Materials exhibiting anti-Stokes shift luminescence have engrossed huge attention in recent years due to their unique ability to convert low energy (high wavelength) excitation photons to high energy (low wavelength) emission photons. This capability arises from the utilization of extra energy from an additional excitation photon leading to a process known as two photon absorption. Owing the unique properties, two/multi-photon absorption has captivated great attention since past few years due to the extensive applications in various fields such as, photodynamic therapy, (Spangler et al., 2004), micro fabrication, (Gonçalves et al., 2017), high-resolution 3D imaging, (Benninger et al., 2008), upconverted lasing, (Medishetty et al., 2017), and 3D optical data storage. (Cumpston et al., 1999). Two/multi-photon absorption processes is of special importance in biomedical application wherein the higher wavelength near infrared light irradiation facilitates deeper penetration into biological tissues and help avoid background fluorescence eventually leading to higher resolution and precise spatial control. Among the few materials that exhibit two/multi-photon absorption, rare earth doped upconversion nanophosphors (UCNPs) have captivated much interest. (Park et al., 2015; Lingeshwar Reddy et al., 2018). Despite their low quantum yield, these nanoparticles have several outstanding qualities, such as sharp emission peaks, large anti-Stokes shift, good photostability, extended lifetimes, no auto-fluorescence and low phototoxicity to biological tissues, (Chen et al., 2014; Zhang et al., 2021), which makes them interesting for potential applications in many fields including nanobiomedicine. (Cheng et al., 2013; Lingeshwar Reddy et al., 2018; Zhang et al., 2020).

While the UCNPs exhibit interesting optical properties, their use in any practical application is governed by the ease of synthesis and the quality of nanocrystals generated. The surface features of the UCNPs is one of the noteworthy quality for practical applications in diverse fields. (Gu et al., 2013; Muhr et al., 2014). Hydrophobic UCNPs can be directly synthesized through conventional methods using capping agents. However, few approaches have been adopted for the synthesis of hydrophilic UCNPs, which can be further classified into 1) two-step approach and 2) one-step approach. Two-step approach is very commonly used where initially a hydrophobic surface is obtained which is further tuned into hydrophilic one though proper ligand modification. (Arppe et al., 2015; Lu et al., 2015; Qin et al., 2016; Vedunova et al., 2016). The reported one-step synthesis approaches can be categorized into three: ligand-mediated, (Cao et al., 2011; Yang et al., 2013), hydrothermal microemulsion (Xiong et al., 2009) and ionothermal syntheses. (Liu et al., 2009; Chen et al., 2010). Mostly, ligand-mediated synthesis generates hydrophilic/hydrophobic UCNPs by using specific ligands such as polymers, or molecules with acid or amino groups, as the surface-capping ligands. (Feng et al., 2006; Liu et al., 2011; Muhr et al., 2014). These functional groups not only make UCNP hydrophilic/hydrophobic but also assist in further functionalization. (Lu et al., 2015; Han et al., 2016).

The most common synthesis approaches are co-precipitation, (Tian et al., 2017), hydrothermal, (Reddy et al., 2021), thermal decomposition, (Ehlert et al., 2008), microwave, (Reddy et al., 2017) etc., which eventually leads to the formation of either hydrophilic or hydrophobic UCNPs. However, hydrophilic or hydrophobic nature of nanophosphors limits their applications. Hence, there is a requirement for the development of a facile synthesis approach to obtain UCNPs that can overcome these shortcomings. Nanoparticles of different surface features are required for application in specified fields. For examples, for biological applications, nanoparticles with hydrophilic surface are favored whereas for various other optoelectronic applications, hydrophobic surfaces are preferred. In this regard, nanoparticles possessing compatible surface features are much beneficial for wider applications. In this work we report a simple approach for the synthesis of ligand free UCNPs thereby trying to overcome some of the shortcomings in conventional UCNP synthesis. In comparison to the above-mentioned methods, ligand-free synthesis can lead to UCNPs possessing nature similar to amphiphilic particles. The as-synthesized nanoparticles show very good upconversion luminescence property under 980 nm near infrared (NIR) light. The strategy could be successfully extended to multiple systems leading to the generation of upconversion luminescence (UCL) covering the entire visible range. These upconversion nanoparticles were also examined for their luminescence in different solvents and pH conditions.

## Experimental Section

### Materials

Yttrium chloride hexahydrate (YCl_3_.6H_2_O), and thulium chloride hexahydrate (TmCl_3_.6H_2_O) were purchased from Sigma. Ytterbium chloride hexahydrate (YbCl_3_.6H_2_O), sodium hydroxide (NaOH), and methanol were purchased from SRL. Ammonium fluoride (NH_4_F), holmium chloride hexahydrate (HoCl_3_.6H_2_O), and erbium chloride (ErCl_3_) were purchased from Alfa Aesar. Calcium chloride (CaCl_2_2H_2_O) was procured from TCI India. Milli Q water was used for all the synthesis and purification stages.

### Ligand-Free Synthesis of NaYF_4_/Yb, Er at Different Reaction Temperatures

NaOH (1 mmol), YCl_3_.6H_2_O (0.78 mmol), YbCl_3_.6H_2_O (0.2 mmol) and ErCl_3_.6H_2_O (0.02 mmol) and NH_4_F (4 mmol) are dissolved separately in minimum amount of Milli Q water. These solutions were then added to 6 ml methanol and water mixture (1:1 ratio, *v/v*) while continues stirring. The solution was allowed to stir for about an hour. The white precipitate was collected and washed with ethanol by means of centrifugation for a minimum of 3 times. The collected white precipitate was kept for overnight drying at 60°C. Further, the solution was divided into five equal portions and heated at different temperatures such as, 100, 200, 400, 600, and 800°C for 3 h to study the effect of temperature.

### Ligand-Free Synthesis of NaYF_4_/Yb, Er at Different Reaction Times

Similar procedure as described above was followed except that the reaction time was varied from 1 to 5 h by keeping the reaction temperature a constant at 600°C.

### Synthesis of Ligand Free NaYF_4_:Yb/Tm and NaYF_4_:Yb/Ho

Similar approach was used for the synthesis of NaYF_4_:Yb/Tm and NaYF_4_:Yb/Ho, the samples were heated at 600°C for 4 h.

### Synthesis of Ligand Free CaF_2_:Yb/Er, CaF_2_:Yb/Ho and CaF_2_:Yb/Tm

Using a similar approach as mentioned above, CaF_2_:Yb/Er (/Ho/Tm) UCNPs were synthesized. The only difference in the synthesis being the use of calcium chloride instead of NaOH and YCl_3_.

### Characterization

The UCL measurements were performed using a Jasco 8500 fluorescence spectrophotometer equipped with a 980 nm laser diode (continuous wave (CW), 500 mW) as the excitation source. Photographic images of colloidal nanophosphors and solid samples were taken using a digital camera without adding any filter. The structural data were collected on a Rigaku Smart Lab 9 kW powder X-ray diffractometer (PXRD) with Ni-filtered Cu Kα irradiation (*λ* = 0.1542 nm) at 45 kV and 100 mA in 2θ ranging from 10° to 90° with a scan rate of 2° per min with a stepping size of 0.02° at room temperature. The morphology of the samples was investigated by using a scanning electron microscope (SEM), FEI Nova Nano SEM-450. The lattice planes were studied using transmission electron microscope (TEM), JEOL JEM-F200 with dual EELS and STEM operating at 200 kV. Energy-dispersive X-ray spectra (EDAX) were obtained using the same TEM instrument. High-resolution X-ray photoelectron spectroscopy (XPS) was carried out using an ESCA Plus spectrometer (Omicron Nanotechnology Ltd. Germany) using a Mg Kα source. The reflectance spectra of the UCNP was collected using Cary series UV-Vis -NIR Spectrophotometer.

## Results and Discussion

**FIGURE 1:**
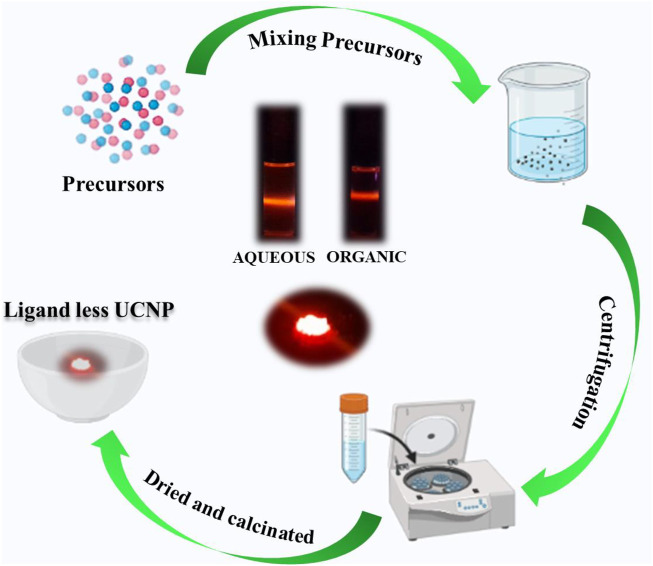
Scheme illustrating the synthesis of ligand free UCNPs.

The synthetic approach adopted for the preparation of ligand free lanthanide doped UCNPs is illustrated in Figure 1. For the synthesis of NaYF_4_:Yb/Er UCNPs, the sensitizer and activator precursors (YCl_3_.6H_2_O, YbCl_3_.6H_2_O and ErCl_3_.6H_2_O) along with other reagents (NH_4_F and NaOH) were mixed thoroughly in methanol-water mixture. No capping ligands, as used in the conventional synthesis procedure, is employed in the present approach. The reaction mixture was subjected to multiple centrifugation and washing using ethanol as solvent to remove excess reagents or impurities. The resulting precipitate was dried followed by calcination at high temperature to obtain UCNPs exhibiting desired properties. Similar approach was adopted for the synthesis of CaF_2_ based UCNPs, namely, CaF_2_:Yb/Er, CaF_2_:Yb/Ho and CaF_2_:Yb/Tm UCNPs are also synthesized.

**FIGURE 2:**
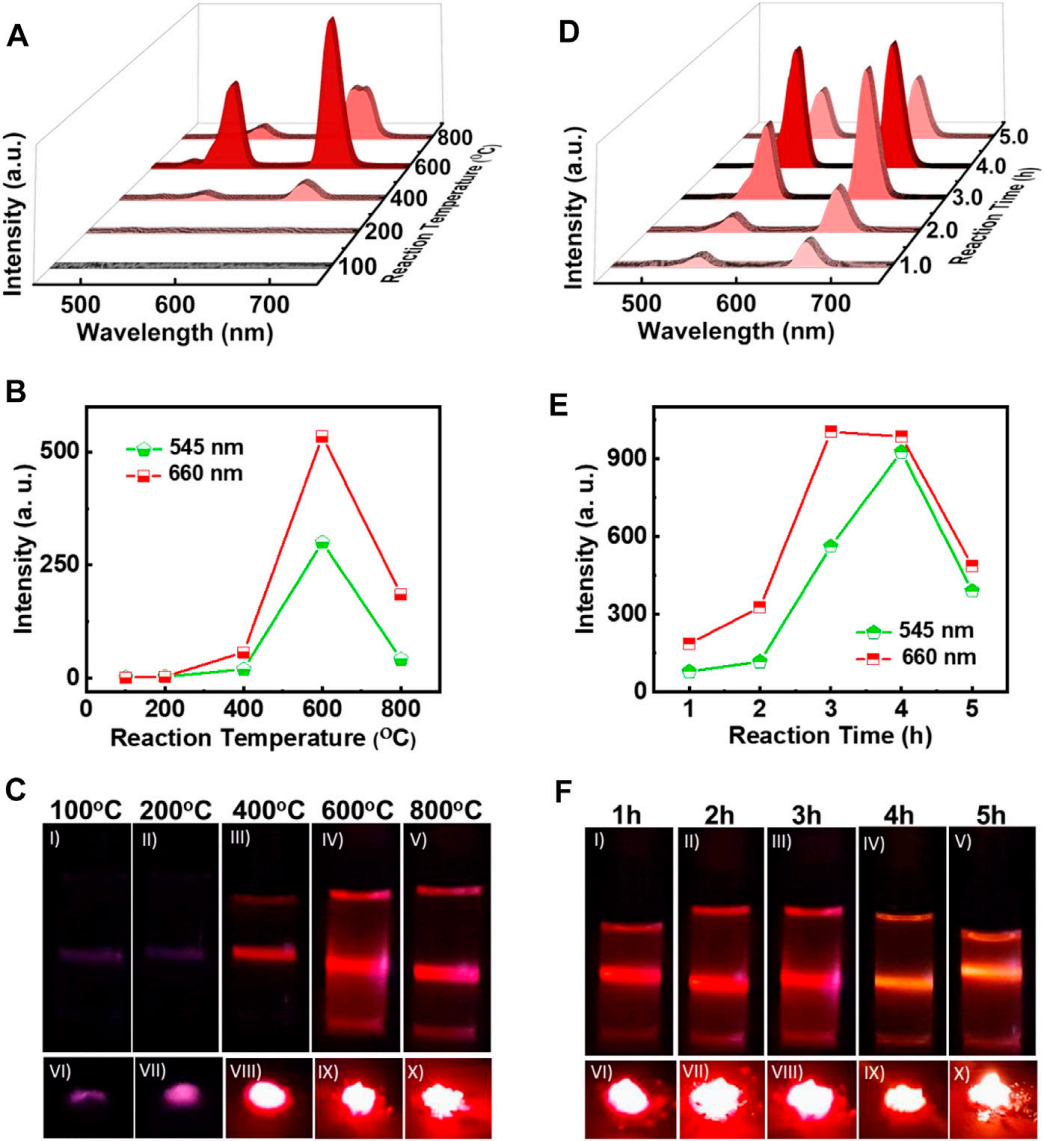
**(A)** UCL spectra, **(B)** plot of intensity variation of green and red emission peaks, and **(C)** photographic images of ligand free NaYF_4_:Yb/Er UCNPs synthesized at different reaction temperatures for a reaction time of 3 h **(D)** UCL spectra, **(E)** plot of intensity variation of green and red emission peaks, and **(F)** photographic images of ligand free UCNPs synthesized for a reaction at 600°C with varying reaction times. Photographic images I-V depicts the colloidal solutions (1 mg/ml in ethanol) and VI-X portrays the solid samples of the UCNPs under 980 nm illumination.

On obtaining a facile protocol for the synthesis of ligand free UCNPs, attempts were made to optimize the reaction conditions to obtain nanophosphors exhibiting enhanced properties. Among the various factors that control the growth of nanophosphors, the calcination temperature plays a crucial role. To standardise the reaction temperature, the synthesis of NaYF_4_:Yb/Er UCNPs was carried out by calcinating the samples at varying temperatures for a period of 3 h. The samples were cooled and then subjected to UV-visible and UCL measurements at room temperature. The reflectance spectra of the solid powder exhibited peak at 980 nm indicating that the UCNPs are doped with the lanthanides (Supplementary Figure S1A). The UCL spectra of the samples dispersed in ethanol collected after excitation using a continuous wave 980 nm laser exhibited two bands at around 546 (green), and 660 nm (red) corresponding to the ^4^S_3/2_ → ^4^I_15/2_, and ^4^F_9/2_ →^4^I_15/2_ transitions respectively of Er^3+^ ions, confirming the formation of the nanophosphors even in the absence of any ligands (Figure 2A, Supplementary Figure S2A). At low temperatures around 100°C no UCL peaks were observed suggesting that no UNCPs were formed at this temperature. Weak peaks started appearing for reaction at 200°C which intensified as the reaction temperature was increased. Most intense peaks were observed for reaction carried out at 600°C. On further increasing the temperature to 800°C, the UCL peaks decreased in intensity and this can be attributed to the defects on the UCNP surface caused due to high temperatures heating. A plot of luminescence intensity corresponding to the ^4^S_3/2_ → ^4^I_15/2_, and ^4^F_9/2_ →^4^I_15/2_ transitions of Er^3+^, vs. reaction temperature showed a gradual increase up to 600°C followed by a decrease in intensity for higher temperature for both the peaks (Figure 2B). The corresponding photographic images of UCNPs samples in solution and solid samples collected upon irradiation with 980 nm CW laser shows no/weak visual emission at low temperature which intensifies with increasing reaction temperature (Figure 2C). Bright red emission could be visualised from both the solution and solid samples synthesised at 600°C. Hence, it could be concluded that a reaction temperature around 600°C is best suited for the synthesis of ligand free NaYF_4_:Yb/Er UCNPs with optimal emission properties.

Another factor that can influence the nucleation and growth of nanocrystals is the reaction time. To investigate the role of reaction time on the synthesis of UCNPs, the duration of calcination was varied from 1 to 5 h. The reaction temperature for all the samples were maintained at 600°C, as optimised earlier. The reflectance spectra of powder samples exhibited peak at 980 nm similar to those observed for samples synthesized at varying temperature, indicating that the nanophosphors are doped with the activator and sensitizer (Supplementary Figure S1B). UCL peaks corresponding to the different transitions in Er^3+^ were observed in all the samples upon excitation using a 980 nm CW laser confirming the formation of lanthanide based UCNPs (Figure 2D). The peak intensities were weak for shorter time periods, and a gradual increase in the peak could be observed with increasing time. A plot of peak intensity for the ^4^S_3/2_ → ^4^I_15/2_, and ^4^F_9/2_ →^4^I_15/2_ transitions of Er^3+^ vs reaction time peaked at 4 h followed by a decrease for longer duration (Figure 2E). The decrease in intensity for reaction carried out for longer time periods could be due to the surface defects caused through prolong heating of samples. The photographic images of solution and solid samples collected upon irradiation with 980 nm CW laser showed low emission at shorter times that intensified with increasing reaction time (Figure 2F). Most intense luminescence was observed for the sample calcinated for around 4 h. Based on the experimental observations, it could be concluded that a reaction temperature of 600°C and a reaction time of 4 h are the optimised conditions required for the synthesis of UCNPs with enhanced properties.

**FIGURE 3:**
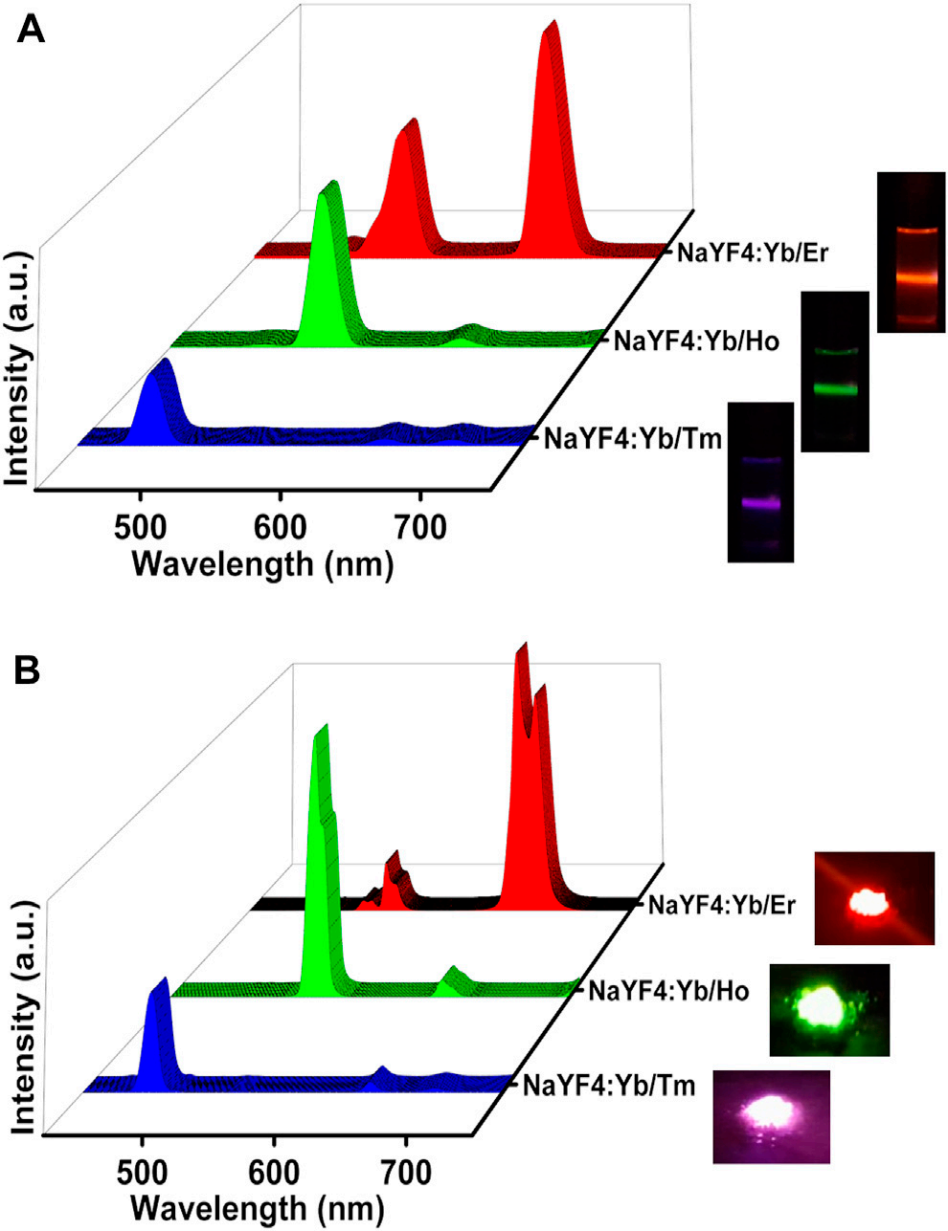
UCL spectra of NaYF_4_:Yb/Tm (blue emitting), NaYF_4_:Yb/Ho UCNPs (green emitting) and NaYF_4_:Yb/Er (red emitting) nanophosphors **(A)** in the solution state and **(B)** in the solid state. The corresponding photographic images of the solution and powder samples upon illumination using a 980 nm torch laser are shown on the right.

After optimizing the reaction temperature and time for the synthesis of red emitting NaYF_4_:Yb/Er UCNPs, the next objective was to rationalise this approach as a general strategy for the synthesis of different class of UCNPs. For investigating the generality of the approach, NaYF_4_:Yb/Tm and NaYF_4_:Yb/Ho UCNPs were synthesized adopting the same approach. The normalized UCL spectra of NaYF_4_:Yb/Tm solution displayed intense peaks at 476 nm corresponding to the ^1^G_4_ → ^3^H_6_ transition of Tm^3+^ ions (Figure 3A, Supplementary Figure S2B). Similarly, the NaYF_4_:Yb/Ho UCNPs exhibited peak at 524 nm corresponding to the ^5^S_2_ → ^5^I_8_ transition in Ho^3+^ ions (Figure 3A,Supplementary Figure S2C). The peak positions at 476 and 524 nm corresponds to the blue and green regions of the electromagnetic spectrum respectively. With the synthesis of three sets of UCNPs we have achieved ligand free synthesis of nanophosphors exhibiting luminescence covering the entire visible range. Moreover, these results prove that the synthesis approach is not limited to one set of nanophosphors but can be adopted as a general approach for the preparation of a wide variety of upconverting luminescent nanoparticles. Similar to the UCNPs in solution, the solid powder samples also displayed intense emission showing promise for these nanophosphors in various material applications (Figure 3B). Likewise, calcium fluoride (CaF_2_) based UCNPs with different activators (Er^3+^, Ho^3+^ and Tm^3+^) were synthesized. Interestingly, these UCNPs also showed good upconversion luminescence confirming that the developed methodology can be considered as universal platform for the synthesis of ligand free fluoride hosted UCNPs (Supplementary Figure S3).

**FIGURE 4:**
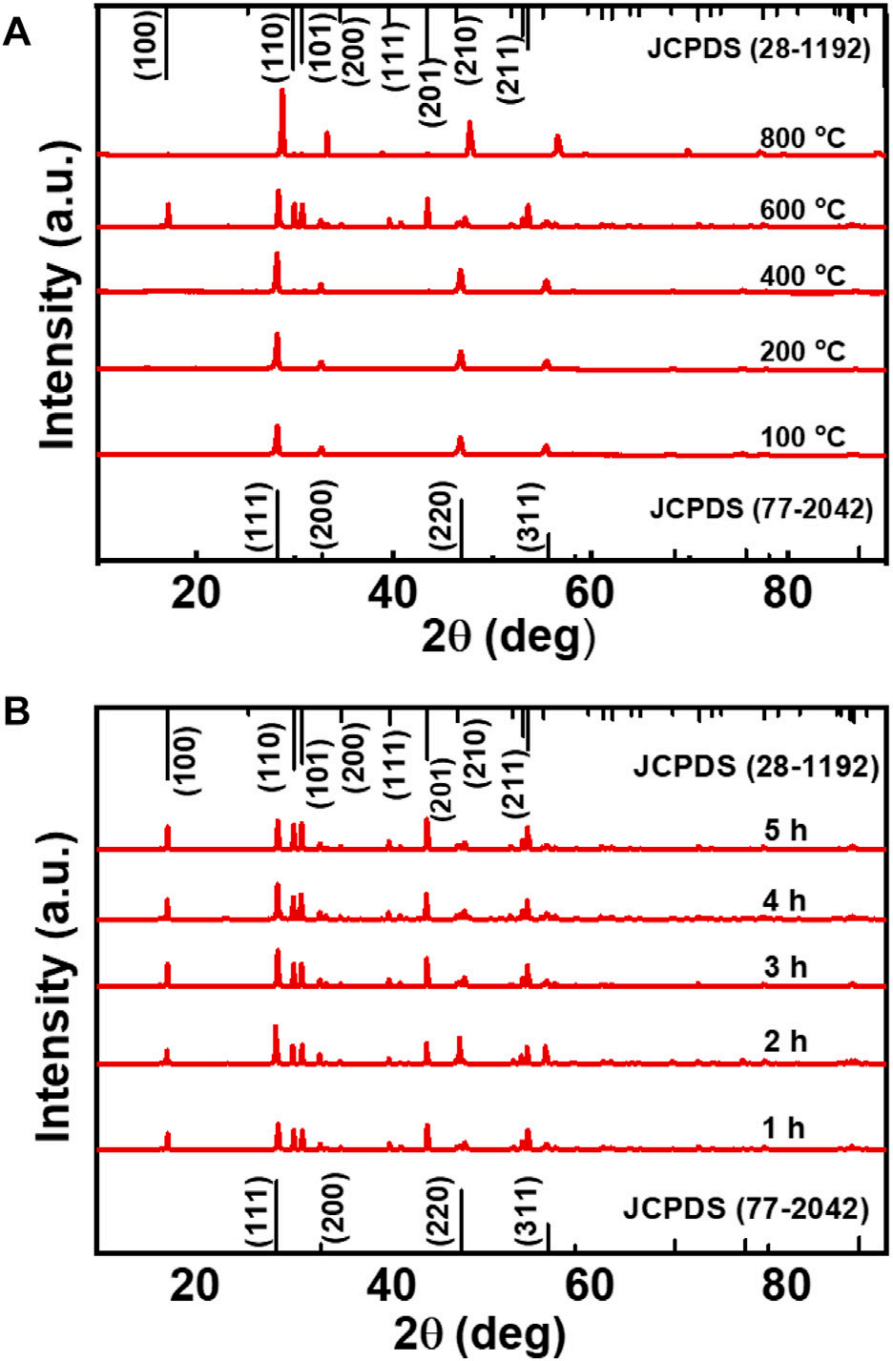
**(A)** XRD spectra of NaYF_4_:Yb/Er UCNPs synthesized at varying reaction temperature (reaction carried out for a period of 3 h), and **(B)** at different reaction time (reaction carried out at a temperature of 600°C).

To analyse the crystalline nature of the samples, powder XRD spectra of all the UCNPs were measured. At low temperature (100°C), NaYF_4_:Yb/Er UCNPs exhibited diffraction peaks at 2θ = 28.09°, 32.57°, 46.72°, and 55.45° which can be attributed to the (111), (200), (220), and (311) planes in cubic phase (JCPDS No. 77–2042). For 100–400°C barely any variation in the lattice phase was seen. However, with increasing temperature from 400°C to 600°C XRD spectra exhibited additional diffraction peaks at 2θ = 17.14°, 30.48°, 30.88°, 34.19°, 40.06°, 43.75°, and 54.65°, attributed to the (100), (110), (101), (200), (111), (201), and (211) planes, respectively of the hexagonal phase (JCPDS No. 28-1192). Hence, a steady increment in the formation of hexagonal phase nanocrystals was observed with increasing temperature. A phase transition from cubic to hexagonal was evident at high temperatures with the hexagonal lattice phase becoming prominent at 600°C (Figure 4A). For heating beyond 600°C, the UCNPs crystallinity transformed back to the cubic phase. The observed phase changes were in agreement with the literature reports on similar systems wherein hexagonal phased upconversion nanocrystals dominates at high temperatures eventually leading to higher UCL. (Wang et al., 2018). Beyond 600°C, the UCNPs possessed only cubic phase crystals and the UCL was also found to be slaked. Hence, a clear phase change could be observed on varying the temperature of the reaction. (Klier and Kumke, 2015). In contrast, the samples synthesized at 600°C by varying the reaction time from 1 to 5 h contained hexagonal and cubic phased nanocrystals in almost an equal ratio (Figure 4B). Similarly, Yb/Tm and Yb/Ho doped NaYF_4_ nanophosphors exhibiting blue and green luminescence respectively, synthesized under optimal reaction conditions (heating at 600°C for 4 h) formed nanocrystals possessing both cubic and hexagonal phases establishing the generality in phase change across different UCNPs (Supplementary Figure S4).

**FIGURE 5:**
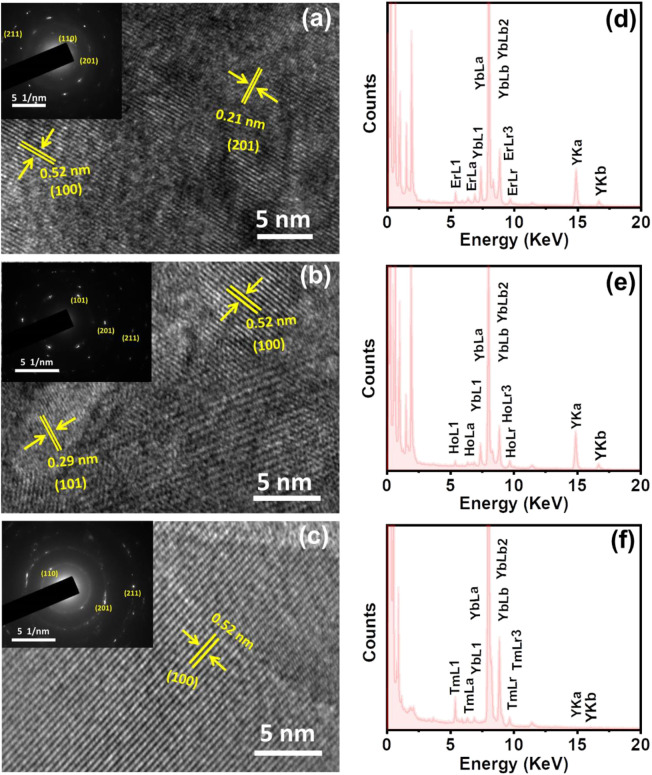
HRTEM images of the ligand free **(A)** NaYF_4_:Yb/Er, **(B)** NaYF_4_:Yb/Ho and **(C)** NaYF_4_:Yb/Tm UCNPs showing the corresponding lattice planes (the nanophosphors are synthesized under optimised reaction conditions discusses above (*vide supra*). The inset in each image shows the corresponding SAED pattern **(D–F)** EDAX spectra of **(D)** NaYF_4_:Yb/Er, **(E)** NaYF_4_:Yb/Ho and **(F)** NaYF_4_:Yb/Tm UCNPs showing the presence of constituent elements in each case.

The morphological features of the ligand free nanophosphors were studied with the help of scanning electron microscopic imaging. The NaYF_4_:Yb/Er nanostructures synthesized under optimised conditions (heating at 600°C for 4 h) showed large micrometer sized particles with irregular morphology (Supplementary Figure S5A). Similar structures were observed with NaYF_4_:Yb/Tm (blue emitting) and NaYF_4_:Yb/Ho UCNPs (green emitting) nanophosphors (Supplementary Figures S5B,C). The average size distribution of UCNPs synthesized using this approach was in the range of 200–500 nm. Further, high-resolution transmission electron microscopy (HR-TEM) was employed to get a deeper insight into the crystalline nature of the nanostructures. HR-TEM images of NaYF_4_:Yb/Er nanophosphors displayed a crystalline nature with lattice distances of 0.52 and 0.21 nm corresponding to a d-spacing for the (100) and (201) lattice planes respectively (Figure 5A). The selected area electron diffraction (SAED) patterns clearly showed the diffraction rings that match with the specific planes for the hexagonal phase of the NaYF_4_:Yb/Er lattice. Similar studies with NaYF_4_:Yb/Ho (green emitting) UCNPs exhibited lattice distances of 0.29 and 0.52 nm corresponding to the d spacing for the (101) and (100) planes (Figure 5B) and that of NaYF_4_:Yb/Tm (blue emitting) exhibited lattice distance of 0.52 nm corresponding to the d spacing of the (100) lattice plane (Figures 5B,C). Diffraction rings observed in the SAED patterns match well with the specific planes for the hexagonal NaYF_4_ lattice (insets in Figures 5B,C). The obtained results were in agreement with the XRD data of the corresponding samples. Further, evidence for the presence of lanthanides (activators and sensitizers) in respective UCNPs was obtained from the EDAX analysis which showed all the constituent elements in the samples (Figures 5D–F). Specifically, the presence of Er, Ho and Tm was confirmed in the red, green and blue emitting UCNPs respectively. This further confirms the presence of dopants as well as activators/sensitizers in the all the three sets of UCNPs synthesized under ligand-free conditions.

**FIGURE 6:**
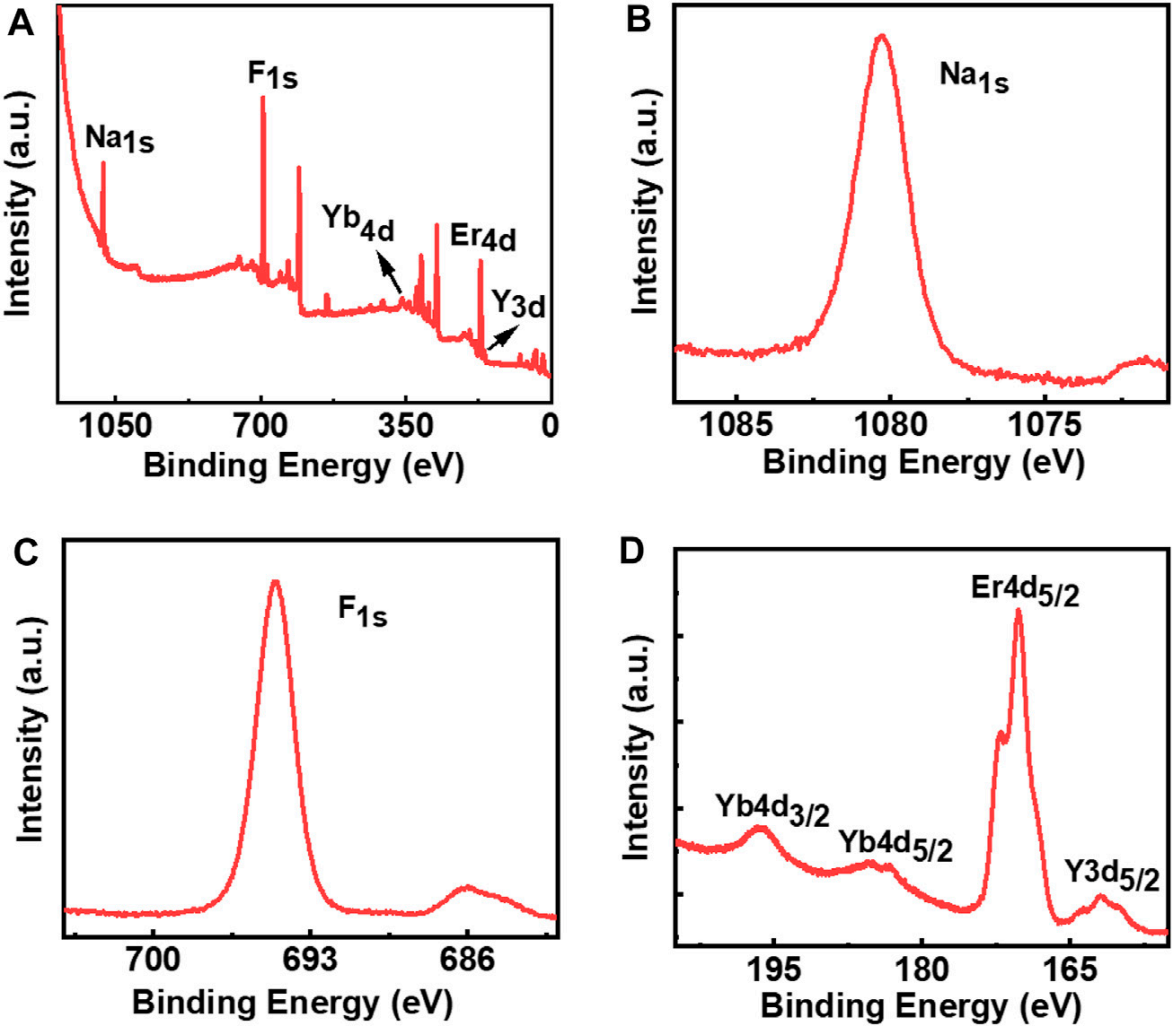
XPS spectra of ligand free NaYF_4_:Yb/Er UCNPs: **(A)** survey spectrum; **(B)** Na 1s; **(C)** F 1s; **(D)** Yb 4d, Er 4d, and Y 3d.

To further confirm the presence of the constituting elements on the surface of the nanophosphors under ligand-free conditions, we have performed the XPS analysis of the three samples. XPS spectra of NaYF_4_:Yb/Er UCNPs synthesized under optimal conditions revealed peaks corresponding to all the constituent elements in the sample. The presence of Na, F, Yb, Er, and Y elements was evident from the XPS survey spectrum (Figure 6A). The peak at 1077.5 can be assigned to the binding energy of Na 1s (Figure 6B) whereas the peak at 691.9 eV corresponds to the binding energy of F 1s (Figure 6C). The peaks observed at 167.8 and 158.7 eV can be attributed to the binding energies of Er 4d and Y 3d, whereas the peaks at 194.5 and 182.6 eV can be assigned to the binding energies of Yb 4d3/2 and Yb 4d5/2, respectively. (Figure 6D). Similarly, the XPS spectra of the NaYF_4_:Yb/Tm (blue emitting) and NaYF_4_:Yb/Ho (green emitting) UCNPs synthesized under optimal conditions exhibited peaks corresponding to the constituent elements in the respective nanophosphors (Supplementary Figures S6,S7). Hence, the XPS data further confirms the presence of all constituent elements in the nanophosphors synthesized under ligand-free conditions.

Having analysed the optical and morphological properties of the nanophosphors, it is important to examine the stability of the nanoparticles under different experimental conditions. The stability of the particles over a longer period of time, under varying temperature, pH and solvent conditions is vital in order to establish the suitability of the nanophosphors for diverse practical applications. Due to the ligand free surface of the as synthesized UCNPs, it would be interesting to study the efficiency of the nanophosphors in different solvents. The ligand-free NaYF_4_:Yb/Er UCNPs were dispersed in different solvents of varying polarity. Remarkably, the UCNPs retained their UCL in both aqueous and organic solvents confirming their consistency in properties in various solvents (Supplementary Figure S8). Moreover, the nanophosphors exhibited good stability in a variety of organic solvents ranging from ethanol, DMF, acetone, dichloromethane, chloroform, tetrahydrofuran and cyclohexane. The stability in conventional ligand capped UCNPs is governed by the nature of the capping ligand. In contrast, the ligand free UCNPs helps stabilise the nanophosphors in diverse solvent environments. Further, the stability of the synthesized UCNPs was analysed under different pH. UCNPs were dispersed in solutions of different pH ranging from 1 to 13 and their UCL spectra were measured upon excitation with 980 nm laser. Almost constant emission intensities (with ±5% error) were observed in acidic, neutral and basic pH (Supplementary Figure S9). The stability was also studied as a function of time. It was observed that the ligand-free UCNPs exhibited good stability over a period of few days for which the UCL was monitored. No noticeable decrease in UCL intensity was observed which confirms that the samples are stable enough to be used for days upon proper storage (Supplementary Figure S10). These analyses of the samples reveal that the synthesized ligand free UCNPs exhibit good stability and the nanophosphors can find suitable application as upconversion luminescent material.

**FIGURE 7:**
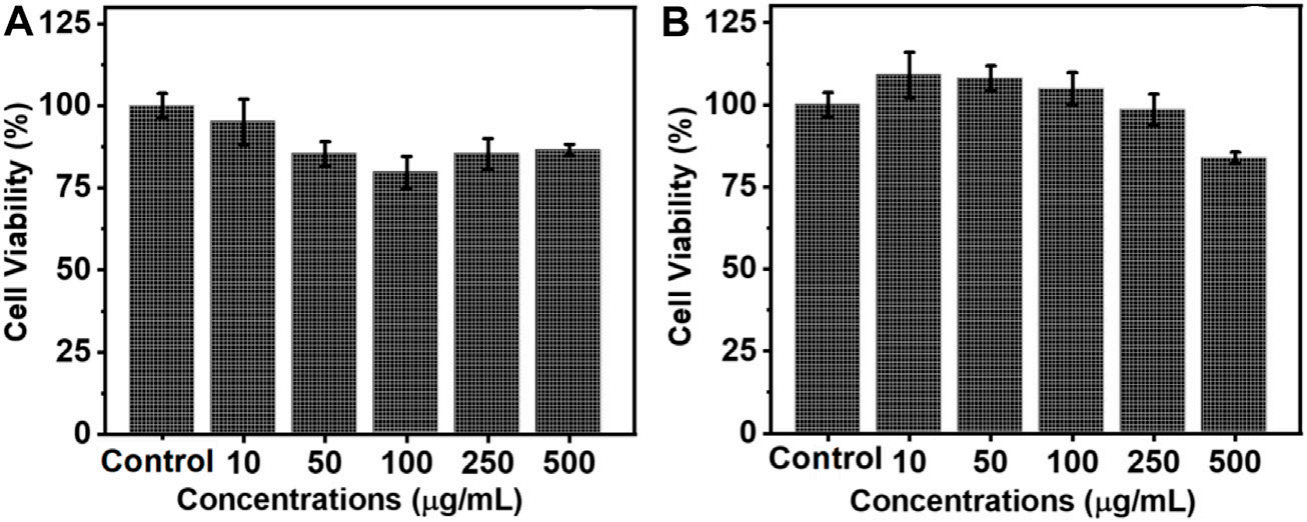
Histogram depicting the cell viability of ovarian cancer cells (A2780) on incubation with varying concentrations of ligand free NaYF_4_:Yb/Er UCNPs after **(A)** 12 and **(B)** 24 h.

For any biological application, it is important that the synthesized nanophosphors are biocompatible. MTT assay is one of the traditional methods employed to analyze the cell viability of the synthesized nanoparticles. To investigate the cell viability, ligand free NaYF_4_:Yb/Er UCNPs of five different concentrations (10, 50, 100, 250 and 500 μg/ml) was treated with ovarian cancer cells (A2780) and the cell death was monitored after 12 and 24 h of incubation. The growth media with no nanoparticles was used as the control. After 12 h of incubation, it was found that the presence of nanoparticles had very little influence on the cells and only a very slight cell death was observed compared to the control sample, which was not incubated with any nanoparticle (Figure 7A). After 24 h a slight enhancement in cell growth compared to the control could be observed for samples incubated with low concentrations of nanoparticles (Figure 7B). This is indicative of the fact that the as synthesized particles do not cause any harmful effect on the growth of the cells. The rate of cell death was minimal even at high concentrations of the nanophosphors incubation confirming that the synthesized particles are biocompatible without any cell toxicity. These results show that the ligand free luminescent upconversion nanoparticles can find potential applications in the biological field, especially as bioimaging agents.

## Conclusion

In summary, we have developed a new approach for the ligand free synthesis of lanthanide based UCNPs. Unlike the conventional methods wherein the UCNPs are capped with organic ligands, the ligand free approach is simple and cost effective. Similar approach was extended to different UCNPs leading to the synthesis of nanophosphors covering the visible range of electromagnetic spectrum. The upconversion luminescence could be observed both in the solution and solid states. The nanocrystals possessed both cubic and hexagonal phase under the optimised conditions of temperature and time. The ligand free nature enabled the UCNPs to be freely dispersed in aqueous as well as organic solvents. Moreover, the synthesized nanoparticles showed stability across the entire pH range and over a reasonably long period of time. The UCNPs exhibited good biocompatibility even at high concentrations establishing the potential of these materials to be used in the biomedical field, and further investigation in this direction are under progress.

## Data Availability

The original contributions presented in the study are included in the article/Supplementary Material, further inquiries can be directed to the corresponding author.
