# Supplementary material for: A Facile Approach for the Ligand Free Synthesis of Biocompatible Upconversion Nanophosphors
Source: Front Chem. 2022 May 31;10:904676. doi: 10.3389/fchem.2022.904676 (PMC9194556; doi:10.3389/fchem.2022.904676)
Supplement: Supplementary file 1 [file DataSheet1.pdf]

Supporting Information

A Facile Approach for the Ligand Free Synthesis of Biocompatible Upconversion Nanophosphors

Elizabeth Shiby,† Kumbam Lingeshwar Reddy,† Jatish Kumar*

(† These authors have contributed equally to this work)

Department of Chemistry, Indian Institute of Science Education and Research (IISER) Tirupati, Tirupati, Andhra Pradesh-517507, India.

**Correspondence: jatish@iisertirupati.ac.in*

Page No.	Contents
S2	Fig. S1: Reflectance spectra of UCNP powder samples
S3	Fig. S2: Scheme illustrating the possible electronic transitions in UCNPs
S4	Fig. S3: UCL spectra of ligand-free CaF_2:Yb/Er (/Ho/Tm) UCNPs
S5	Fig. S4: Powder XRD spectra of blue, green and red emitting UCNPs
S5	Fig. S5: SEM images of UCNPs
S6	Fig. S6: XPS spectra of $NaYF_4$:Yb/Tm UCNPs
S7	Fig. S7: XPS spectra of $NaYF_4$:Yb/Ho UCNPs
S8	Fig. S8: UCL spectra of ligand free UCNPs in different solvents
S8	Fig. S9: UCL spectra of ligand free UCNPs monitored at different pH
S9	Fig. S10: UCL spectra of ligand free UCNPs monitored as a function of time

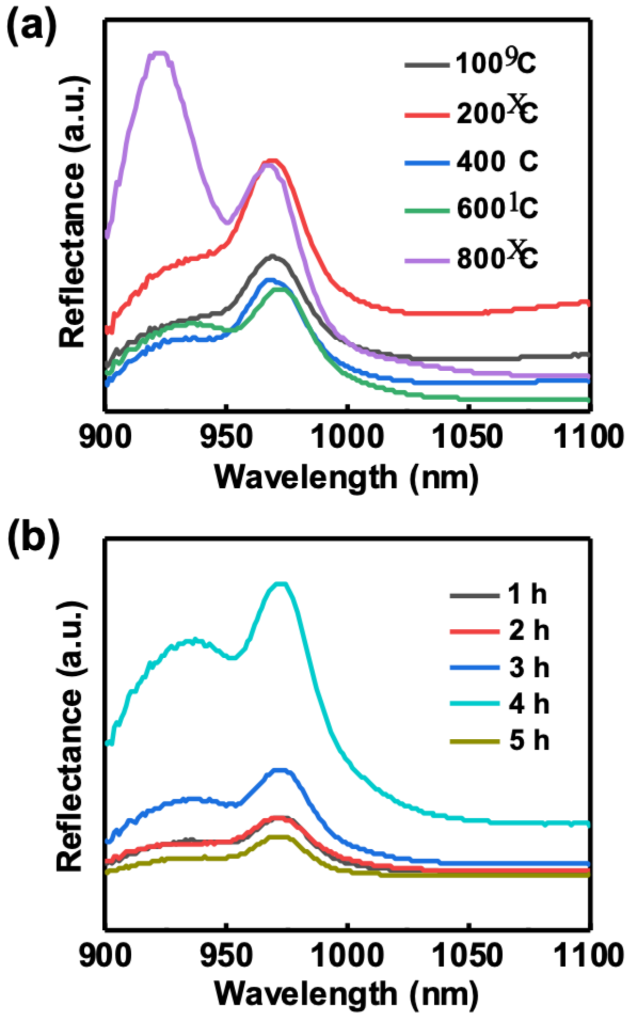

Figure S1. Reflectance spectra of $NaYF_4$:Yb/Er UCNPs synthesized under (a) varying reaction temperature (for a period of 3 h) and a reaction time of 4 h (at a reaction temperature of 600 ^{o}C).

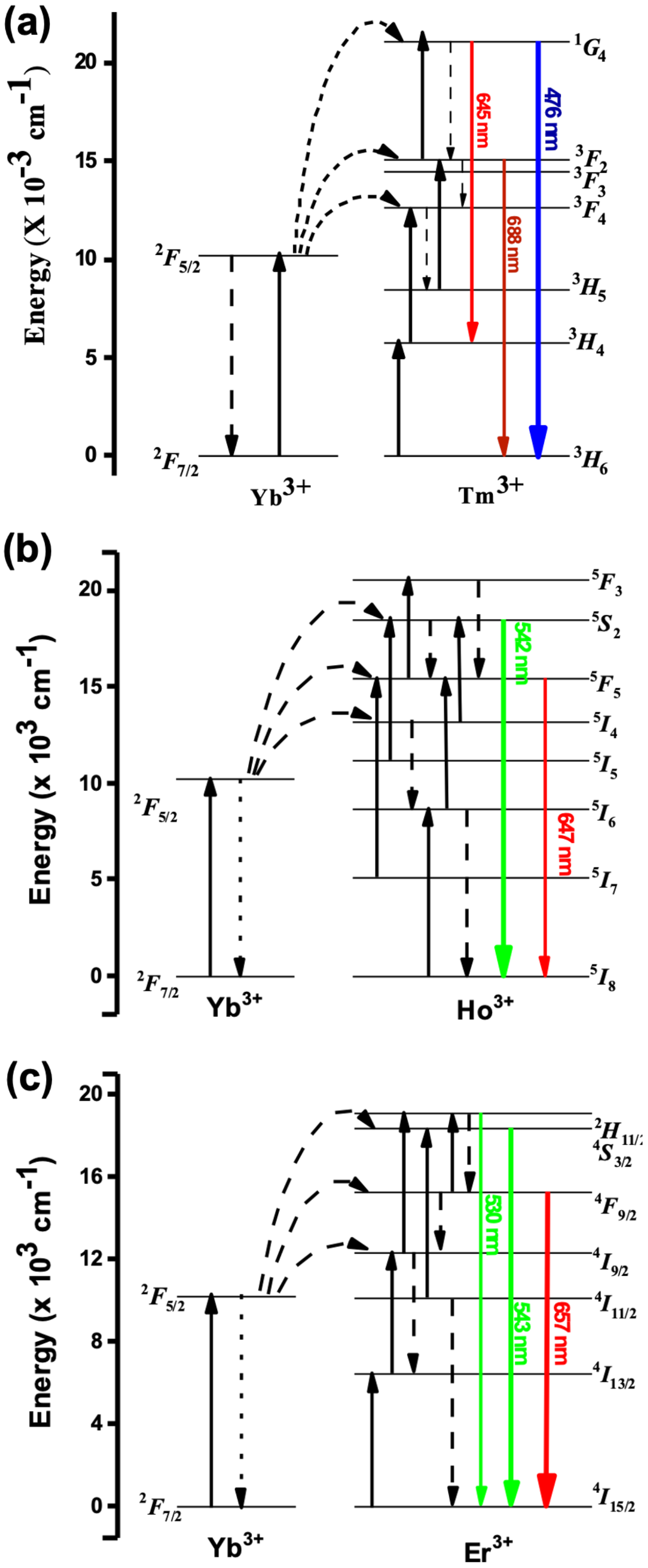

Figure S2. Scheme illustrating the possible electronic transitions in (a) $NaYF_4$:Yb/Tm, (b) $NaYF_4$:Yb/Ho (green) and (c) $NaYF_4$:Yb/Er (red) leading to the corresponding emission bands. The prominent transitions in each case are marked using thick arrows.

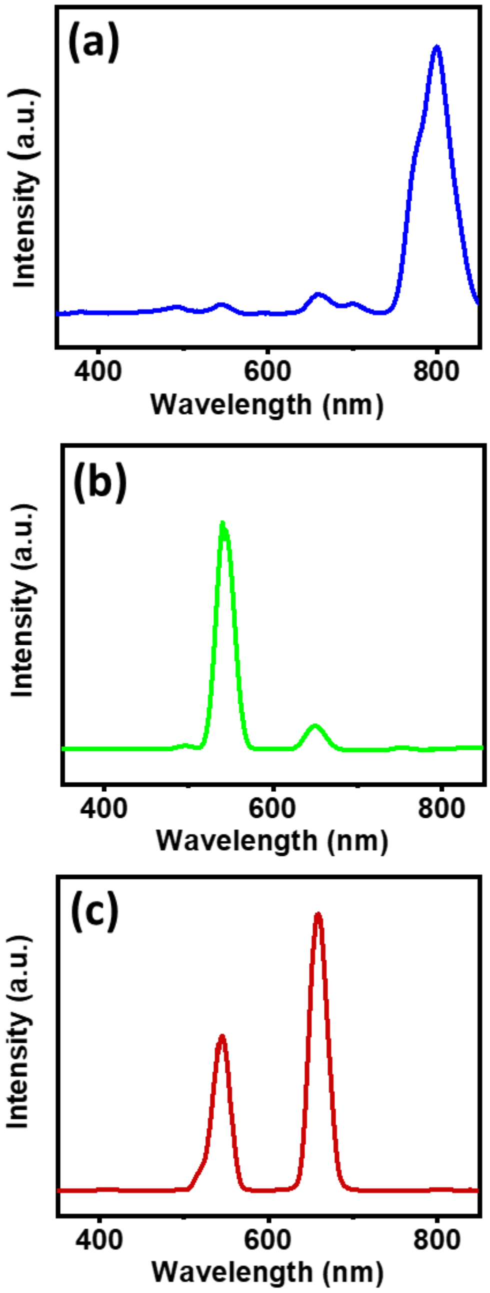

Figure S3. UCL spectra of ligand-free a) CaF_2:Yb/Tm, b) CaF_2:Yb/Ho and c) CaF_2:Yb/Er UCNPs

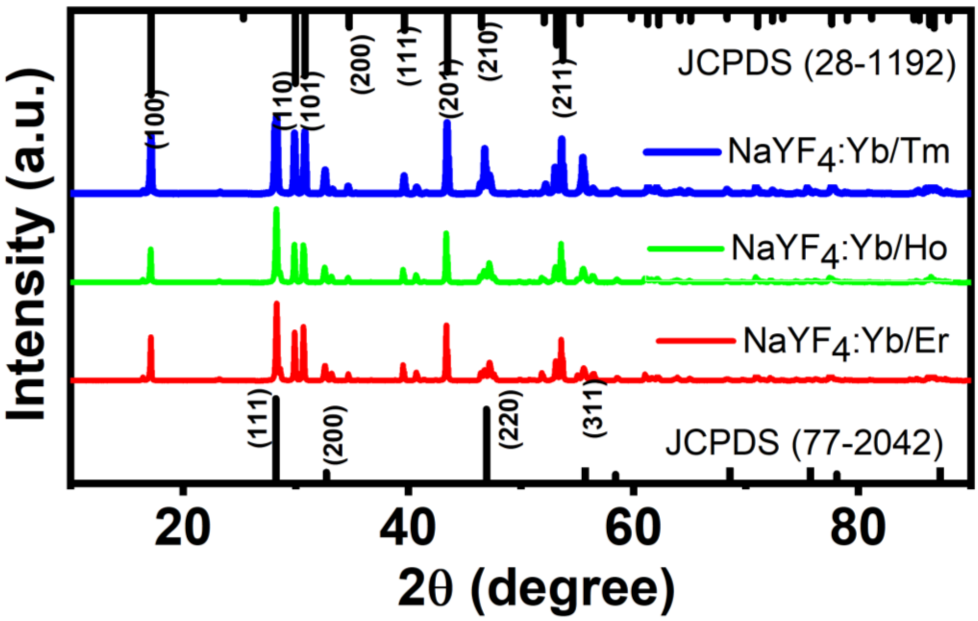

Figure S4. Powder XRD spectra of $NaYF_4$:Yb/Tm (blue), $NaYF_4$:Yb/Ho (green) and $NaYF_4$:Yb/Er (red) UCNP synthesized at a temperature of 600 ℃ for a reaction carried out for a time period of 4 h.

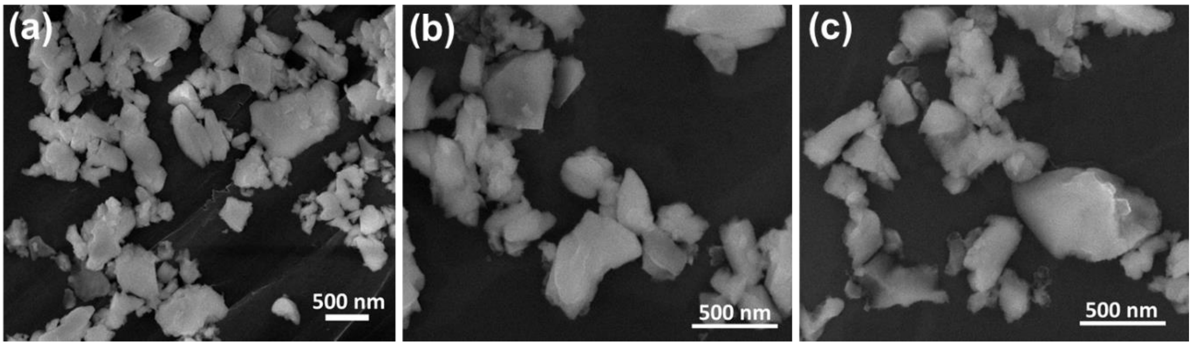

Figure S5: SEM images of (a) $NaYF_4$:Yb/Er, (b) $NaYF_4$:Yb/Ho, and (c) $NaYF_4$:Yb/Tm UCNPs synthesized under optimised conditions (calcination at 600 ^{o}C for 4 h).

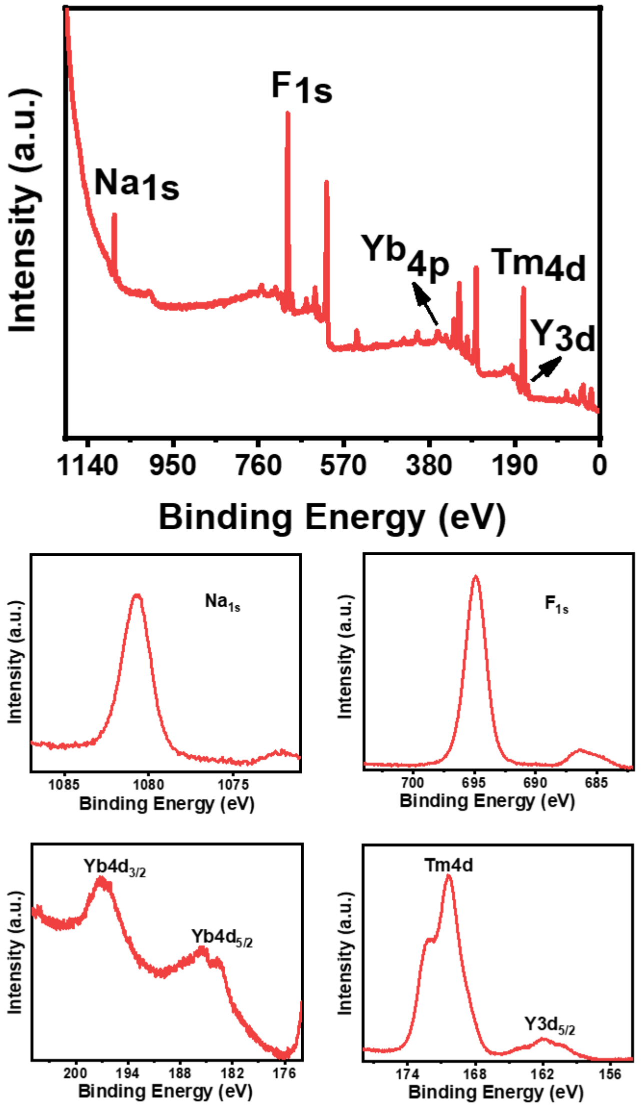

Figure S6. XPS spectra of $NaYF_4$:Yb/Tm UCNPs: (a) survey spectrum; (b) Na 1s; (c) F 1s; (d) Yb 4d, and (e) Tm 4d, and Y 3d.

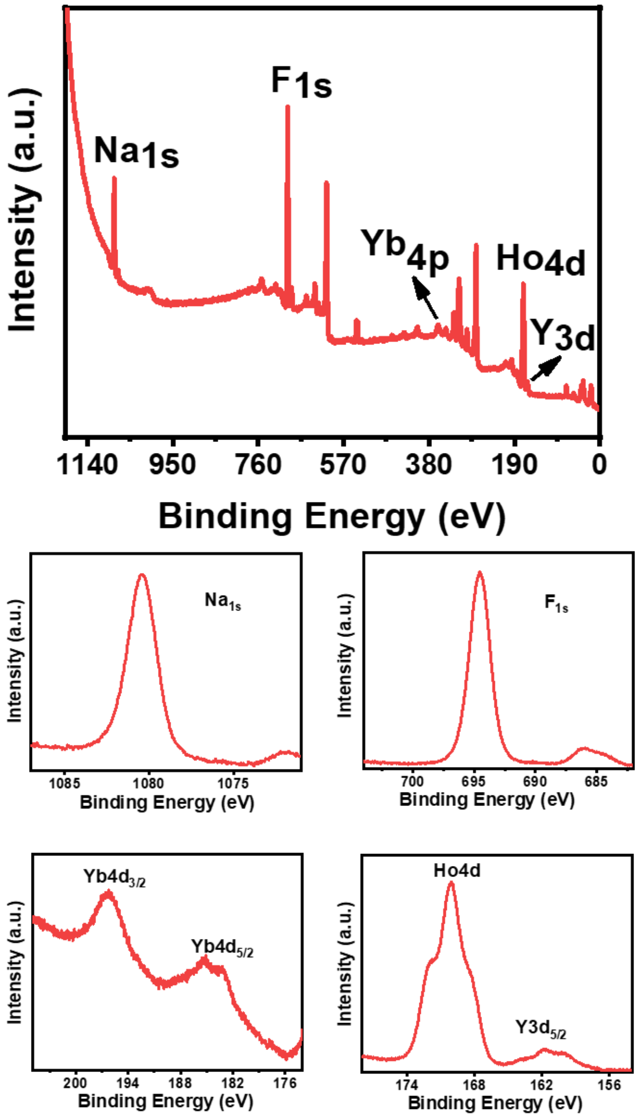

Figure S7. XPS spectra of $NaYF_4$:Yb/Ho UCNPs: (a) survey spectrum; (b) Na 1s; (c) F 1s; (d) Yb 4d, and (e) Ho 4d, and Y 3d.

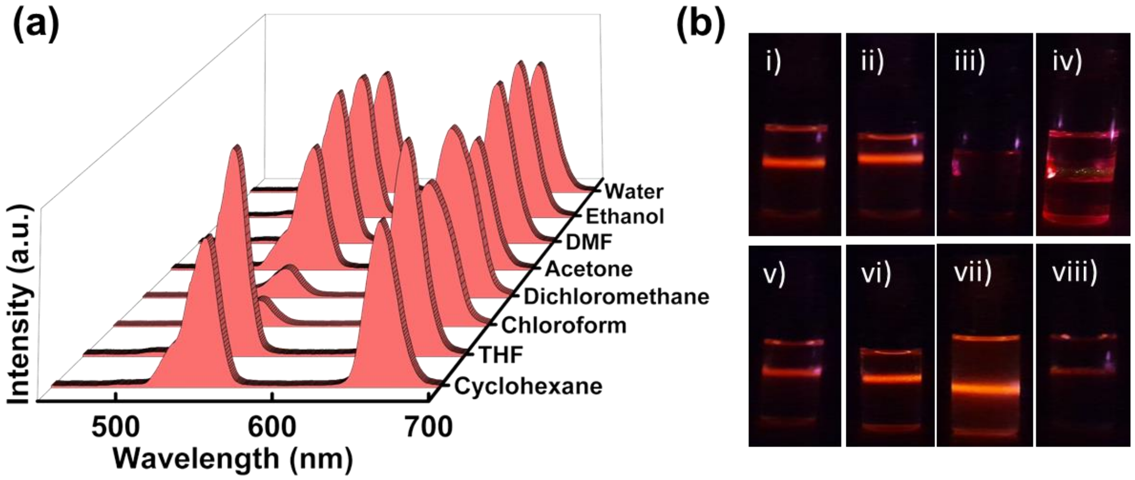

Figure S8. (a) UCL spectra of ligand free $NaYF_4$:Yb/Er UCNPs in different solvents and (b) the corresponding photographic images collected after illumination with 980 nm CW laser. (i) cyclohexane, (ii) THF, (iii) chloroform, (iv) DCM, (v) acetone, (vi) DMF, (vii) ethanol and (viii) water.

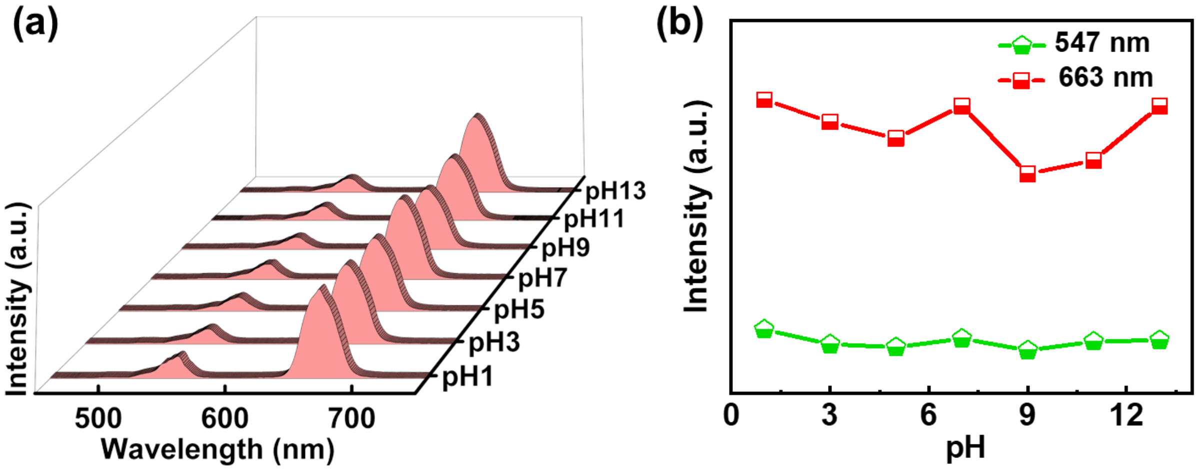

Figure S9. (a) UCL spectra of ligand free $NaYF_4$:Yb/Er UCNPs monitored at different pH. (b) A plot of pH vs intensity for the red and green emitting peaks.

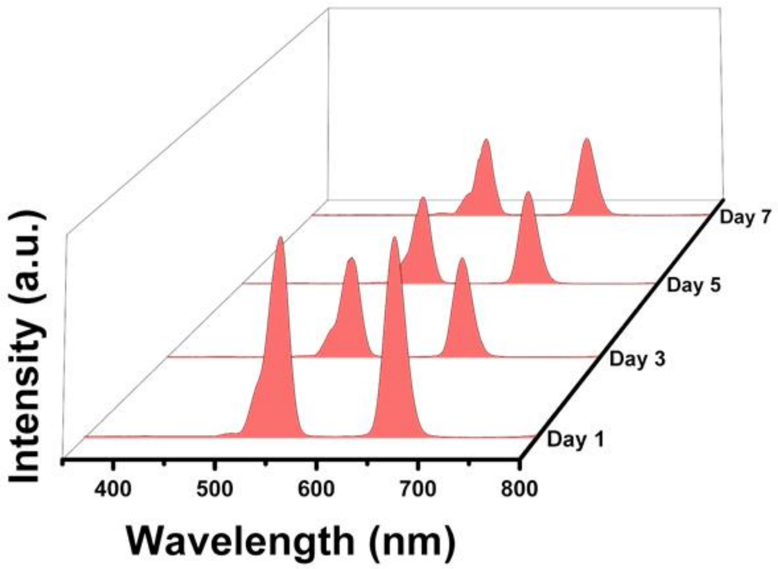

Figure S10. (a) UCL spectra of ligand free $NaYF_4$:Yb/Er UCNPs monitored as a function of time.
